# Using Diffusion Tensor Imaging to Evaluate Microstructural Changes and Outcomes after Radiofrequency Rhizotomy of Trigeminal Nerves in Patients with Trigeminal Neuralgia

**DOI:** 10.1371/journal.pone.0167584

**Published:** 2016-12-20

**Authors:** Shu-Tian Chen, Jen-Tsung Yang, Mei-Yu Yeh, Hsu-Huei Weng, Chih-Feng Chen, Yuan-Hsiung Tsai

**Affiliations:** 1 Department of Diagnostic Radiology, Chang Gung Memorial Hospital at Chiayi, Chiayi, Taiwan; 2 Department of Neurosurgery, Chang Gung Memorial Hospital at Chiayi, Chiayi, Taiwan; 3 Department of Diagnostic Radiology, Asia University Hospital, Taichung, Taiwan; Universita degli Studi di Palermo, ITALY

## Abstract

Trigeminal neuralgia is characterized by facial pain that may be sudden, intense, and recurrent. Our aim was to investigate microstructural tissue changes of the trigeminal nerve in patients with trigeminal neuralgia resulting from neurovascular compression by diffusion tensor imaging, and to test the predictive value of diffusion tensor imaging for determining outcomes after radiofrequency rhizotomy. Forty-three patients with trigeminal neuralgia were recruited, and diffusion tensor imaging was performed before radiofrequency rhizotomy. By selecting the cisternal segment of the trigeminal nerve manually, we measured the volume of trigeminal nerve, fractional anisotropy, apparent diffusion coefficient, axial diffusivity, and radial diffusivity. The apparent diffusion coefficient and mean value of fractional anisotropy, axial diffusivity, and radial diffusivity were compared between the affected and normal side in the same patient, and were correlated with pre-rhizotomy and post-rhizotomy visual analogue scale pain scores. The results showed the affected side had significantly decreased fractional anisotropy, increased apparent diffusion coefficient and radial diffusivity, and no significant change of axial diffusivity. The volume of the trigeminal nerve on affected side was also significantly smaller. There was a trend of fractional anisotropy reduction and visual analogue scale pain score reduction (P = 0.072). The results suggest that demyelination without axonal injury, and decreased size of the trigeminal nerve, are the microstructural abnormalities of the trigeminal nerve in patients with trigeminal neuralgia caused by neurovascular compression. The application of diffusion tensor imaging in understanding the pathophysiology of trigeminal neuralgia, and predicting the treatment effect has potential and warrants further study.

## Introduction

Trigeminal neuralgia (TN) is a common cause of facial pain, and is characterized by recurrent sudden onset, unilateral, brief electric shock-like pain that is localized to the sensory supply area of trigeminal nerve, i.e., cranial nerve V (CN V) [1]. Neurovascular compression (NVC) of the CN V at the root entry zone (REZ) is the most common cause of TN [2, 3], and can be diagnosed by 3D-gradient echo sequence magnetic resonance imaging (MRI) [4]. TN is usually treated with anticonvulsants, microvascular decompression, stereotactic radiosurgery, or minimally invasive percutaneous lesioning of CN V such as radiofrequency rhizotomy [5, 6]. However, the pathophysiology of TN is still debated. Postoperative histopathological studies have shown axonal atrophy and demyelination in patients with TN [2, 7, 8]. Several recent studies have revealed that diffusion tensor imaging (DTI) enables identification of microstructural abnormalities, including decreased fractional anisotropy (FA) and increased radial diffusivity (RD) [9–14].

FA reduction has been shown to be correlated with visual analogue scale (VAS) pain scores in patients with TN, which suggests that DTI metrics could be a MRI marker for monitoring clinical severity [13]. However, the correlation of DTI metrics and prognosis after intervention has never been reported. The aim of this study was to investigate microstructural tissue changes of CN V in patients with TN resulting from NVC by multiple DTI metrics, and correlate the DTI metrics with outcome after radiofrequency rhizotomy.

## Materials and Methods

### Participants

**Fig 1:**
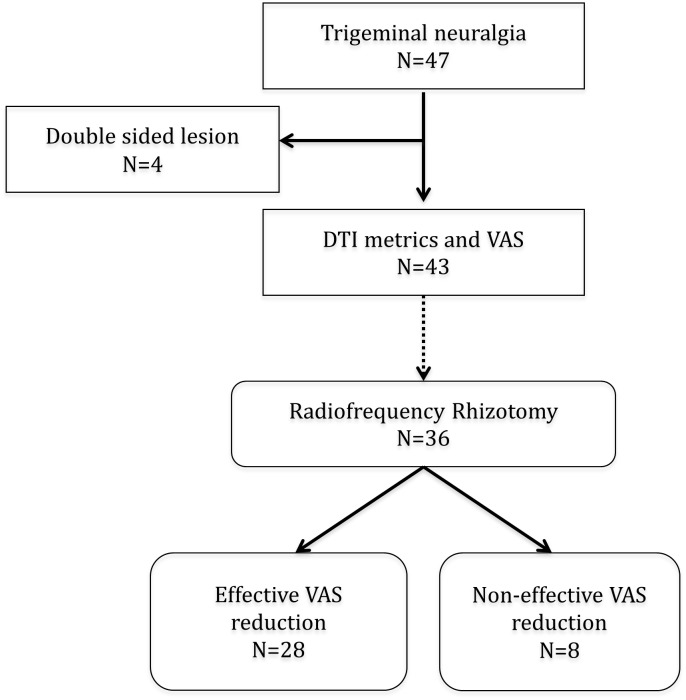
Flow diagram of patient selection.

Forty-seven patients with TN were prospectively enrolled into this study. All patients were diagnosed with TN according to the criteria of the International Headache Society for TN, and underwent MRI. Four patients had history of TN on the contralateral side, and were excluded from further the analysis. Among the 43 patients with unilateral TN, 36 (83.7%) received radiofrequency rhizotomy after MRI. VAS pain scores were assessed twice, before rhizotomy (pre-rhizotomy VAS) and 1 month after rhizotomy (post-rhizotomy VAS). Effective responders were defined as patients with VAS reduction [(post-rhizotomy VAS)—(pre-rhizotomy VAS) ≥ 6] (Fig 1).

### MRI acquisition and processing

**Fig 2:**
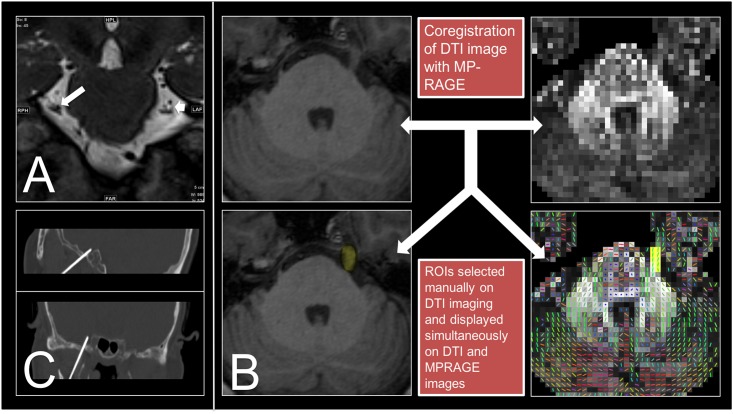
Images of trigeminal neuralgia, image processing steps, and rhizotomy. (A) Coronal T2-weighted image showed CN V was compressed by the superior cerebellar artery on the affected side (long arrow), compared with the normal unaffected side (short arrow) in a 55 year-old women with TN. (B) Co-registration of diffusion tensor image with MP-RAGE image in the axial plane, and selection of voxels covering the largest cisternal segment of the trigeminal nerve on co-registered image. (C) Percutaneous CT-guided radiofrequency rhizotomy of the right side (affected side) of the Gasserian ganglion was performed one day after MRI scanning.

All data were collected with a 3 Tesla Siemens Verio MRI system (Siemens Medical System, Erlangen, Germany) using a 32-channel head coil. DTI sequences were obtained using a readout-segmented echoplanar imaging (RS-EPI) sequence (Syngo RESOLVE; Siemens Medical System) with the following parameters: matrix size = 110 × 110; FOV = 220 mm; section thickness = 2 mm; readout segments = 5; slice = 50 without gap; b value = 0 and 1,000 s/mm^2^; diffusion directions = 30; TR = 6700 ms; TE1/TE2 = 70/95 ms; spatial resolution = 2 mm × 2 mm × 2 mm; echo spacing = 0.32 ms; echo reading time = 7.04 ms; acquisition time = 20 minutes 58 seconds. 3D MP-RAGE anatomical images were obtained using a gradient echo sequence with the following parameters: TR = 1900 ms; TE = 2.98 ms; FOV = 230 mm; matrix = 220 × 256; slice number = 160; spatial resolution = 0.9 mm × 0.9 mm × 0.9 mm; acquisition time = 5 minutes 59 seconds. The DSI Studio software package utilities (http://dsi-studio.labsolver.org/) was used for the post-processing of DTI data. The DTI maps were co-registered to the 3D MP-RAGE anatomical images in the axial plane. Then, the regions of interest (ROIs) were placed on the co-registered image and at the slice that had the largest voxel numbers at the cistern segment of CN V. All imaging voxels covering the cisternal segment of CN V were selected manually on the DTI images by an experienced neuroradiologist (YH Tsai) who was blinded to patient data. During selection, the ROIs were displayed simultaneously on the MP-RAGE images in order to check the accuracy of ROI locations (Fig 2). The average DTI metrics of all voxels within the ROI, including apparent diffusion coefficient (ADC), fractional anisotropy (FA), axial diffusivity (AD), and radial diffusivity (RD), were then calculated. The volume of CN V on MP-RAGE images was estimated using NIH ImageJ software (https://imagej.nih.gov/ij/). To test the inter-observer reliability, another experienced neuroradiologist (CF Chen) who was also blinded to patient data repeated the measurements on the affected CN V.

### Radiofrequency rhizotomy

Percutaneous CT-guided radiofrequency rhizotomy was performed by an experienced neurosurgeon. The rhizotomy needle was inserted into the location confirmed by reproduction of paresthesia upon stimulation, covering the distribution of a specific division of CN V. The lesion at the Gasserian ganglion was produced by radiofrequency thermocoagulation (Radionics, Inc. Burlington, MA, USA) at 60°C for 60 seconds.

### Statistical analysis

All DTI metrics, including ADC, FA, AD, and RD, were tested for normality of distribution using the Kolmogorov-Smirnov test. The mean values of DTI metrics were compared between the affected side and the contralateral side in the same patient by using a two-tailed t test, as were the mean DTI metrics of responders and non-responders. The inter-observer reliability between two neuroradiologists for measuring FA of the affected CN V were tested with two-way intra-class correlation coefficients (ICC) with absolute agreement. Values of P < 0.05 were considered to indicate a significant difference. All statistical calculations were performed with SPSS V.18 software (SPSS, Chicago, IL).

## Results

Patient characteristics are summarized in Table 1. A total of 43 patients were included (16 males, 27 females, mean age 58.8 ± 11.0 years), and the left side was affected in 19 patients, and right side in 24 patients. The mean time interval between MRI scan and radiofrequency rhizotomy was 7.9 days.

**Table 1:** Patient characteristics.

Characteristic	Number (percentage) or Mean (SD)
**Total number of patients**	43
**Age, y**	58.8 (11.1)
**Male gender**	16 (37.2%)
**Left side**	19 (44.2%)
**Pain duration, mo**	65.2 (65.8)
**Interval between MRI and Radiofrequency rhizotomy, d**	7.9 (8.8)
**Numbers of patient that received radiofrequency rhizotomy**	36 (83.7%)
**VAS pain score**	
**Pre-radiofrequency rhizotomy**	9.4 (0.9)
**Post-radiofrequency rhizotomy**	1.7 (2.0)

**Fig 3:**
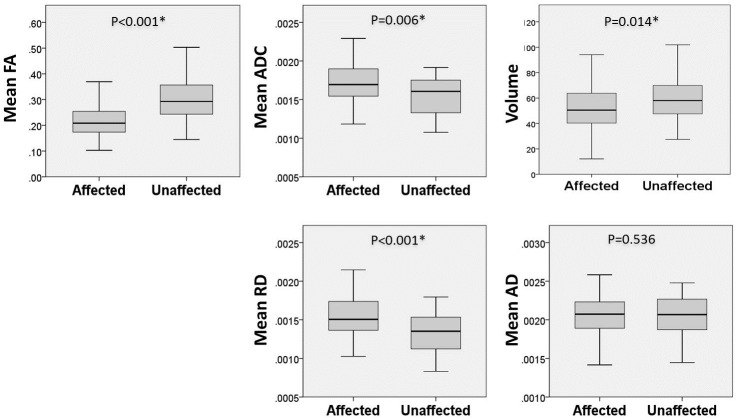
Box-and-whisker plots of DTI metrics between the affected and contralateral TN. Box-and-whisker plots demonstrating medians and interquartile ranges (25–75 percentiles) of fractional anisotropy (FA), apparent diffusion coefficient (ADC), radial diffusivity (RD), axial diffusivity (AD), and volume. An outliner was removed in advance. (*P < 0.05)

Differences in DTI metrics between the affected side and contralateral side are shown in Table 2. The volume of the affected CN V (51.2 ± 20.2 mm^3^) was significantly smaller than that of the contralateral nerve (62.0 ± 19.8 mm^3^) (P = 0.014). FA was significantly lower on the affected side (0.216 ± 0.073) as compared to the unaffected side (0.313 ± 0.106) (P < .001). ADC and RD were significantly greater on the affected side (1.70 ± 0.27 ×10^−3^ mm^2^/s and 1.53 ± 0.28 × 10^−3^ mm^2^/s, respectively) as compared to the contralateral unaffected side (1.54 ± 0.28 × 10^−3^ mm^2^/s; 1.30 ± 0.29 × 10^−3^ mm^2^/s, respectively) (P = 0.006 and < 0.001, respectively). There was no significant difference in AD between the affected (2.05 ± 0.29 × 10^−3^ mm^2^/s and uninvolved contralateral side (2.01 ± 0.32 × 10^−3^ mm^2^/s) (P = 0.536) (Table 2 and Fig 3). The ICC showed high inter-observer reliability for measurement of the affected CN V FA (average measures ICC = 0.898).

**Table 2:** Differences in DTI metrics between the affected and contralateral TN (N = 43).

	Lesion Mean (SD)	Normal Mean (SD)	P value
**Volume (mm**^**3**^**)**	51.2 (20.2)	62.0 (19.8)	0.014*
**Fractional anisotropy**	0.216 (0.073)	0.313 (0.106)	< 0.001*
**Apparent diffusion coefficiency (×10**^**−3**^**)**	1.703 (0.270)	1.536 (0.278)	0.006*
**Axial diffusivity (×10**^**−3**^**)**	2.052 (0.295)	2.012 (0.318)	0.536
**Radial diffusivity (×10**^**−3**^**)**	1.528 (0.281)	1.298 (0.288)	< 0.001*

* P < 0.05 was considered to indicate a significant difference.

**Fig 4:**
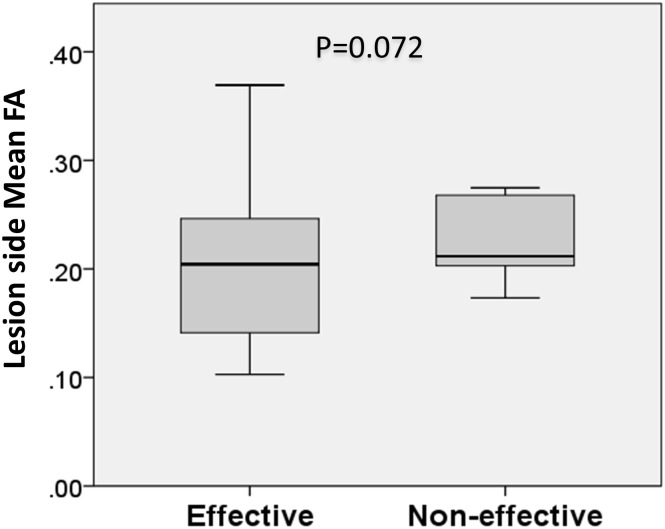
Box-and-whisker plots of fractional anisotropy between effective responders and non-responders. Box-and-whisker plots demonstrating medians and interquartile ranges (25–75 percentiles) of fractional anisotropy. An outliner was removed in advanced.

There were no statistical differences in DTI metrics and volume between effective responders and non-responders. However, there was a trend of FA reduction and VAS pain score reduction (P = 0.072) (Table 3 and Fig 4).

**Table 3:** DTI metrics associated with patient response to RFA (N = 36).

	Effective responders (n = 28) Mean (SD)	Non-responders (n = 8) Mean (SD)	P value
**Volume (mm**^**3**^**)**	51.83 (19.0)	43.13 (21.78)	0.277
**Fractional anisotropy**	0.202 (0.069)	0.256(0.086)	0.072
**Apparent diffusion coefficiency (×10**^**−3**^**)**	1.729 (0.288)	1.662 (0.228)	0.552
**Axial diffusivity (×10**^**−3**^**)**	2.069 (0.327)	2.044 (0.229)	0.842
**Radial diffusivity (×10**^**−3**^**)**	1.559 (0.292)	1.471 (0.261)	0.450

## Discussion

This study examined microstructural abnormalities of CN V in patients with TN due to NVC by multiple DTI metrics. Compared with the unaffected side, the affected side showed significantly decreased FA, increased ADC and RD, and no significant change of AD. The volume of CN V on the affected side was significantly smaller than on the unaffected side. The results suggest that the pathological features of TN are prominently demyelination and decreased CN V size.

In this study, the lower FA of CN V at REZ of the affected side compared to the unaffected side was due to an increase in RD, without significant difference in AD. These changes of DTI metrics may be the consequences of demyelination, edema, and also decreased axonal packing density. Although both demyelination and edema may cause a decrease in FD and an increase in RD, we also found an increased ADC on the affected side, which indicated vasogenic edema and implies increased extracellular space in view of the edematous effect. This is contrary to our finding that the volume of CN V of the affected side is smaller than that of the unaffected side. Therefore, we contribute the changes in DTI metrics in our study to demyelination rather than edema.

AD and RD are believed to be useful tools to differentiate axonal injury and demyelination, and our results suggest that demyelination plays a more significant role than atrophy in microstructural changes of CN V. This result is consistence with that of previous studies [13, 14]. Myelin is thought to inhibit ephaptic interactions [15]. The consequences of demyelination are (1) altered action potential and spontaneous activity, (2) current leakage due to loss of the insulating effects of myelin, and (3) ephaptic spread of excitation [16]. Therefore, a focal demyelinating lesion may be a source of spontaneous activity, and further spread the excitation laterally to fibers that are supposed to be electrically silent. Our finding is consistent with the hypothesis that ephaptic transmission caused by focal demyelination of the CN V root is the cause of TN [17, 18].

We found that the volume of CN V of the affected side was smaller than that of the unaffected side. Erbay et al. [19] reported similar results, and they believed that CN V atrophy is the reason for the pain of TN. Our results suggest demyelination, rather than axonal injury, is the major cause of CN V atrophy due to neurovascular compression, as the results of a pathological study by Hilton et al. [7] reported.

To the best of our knowledge, there have been no previous studies using DTI metrics as an imaging tool to predict the effect of TN treatment. The VAS is a simple and reproducible research tool for the assessment of pain severity [20, 21]. In our study, VAS pain scores were a mean of more than 9 points before radiofrequency rhizotomy, and decreased to a mean of 1.7 points 1 month after treatment. Radiofrequency rhizotomy treatment of TN is based on the fact that Aδ and C fibers are more sensitive to thermocoagulation than Aα and β fibers [22, 23]. Irreversible damage to small, unmyelinated pain fibers blocks pain sensation without sensory and motor nerve damage when the temperature is 55 to 70°C [24]. We found a trend of FA reduction and VAS pain score reduction, but the result did not reach statistical significance. This may suggest that the more severe the microstructural abnormalities of the trigeminal nerve, the greater the sensitivity to the thermocoagulation effect on pain fibers. Conversely, less FA reduction may mean relative preservation of nerve integrity, and that a higher temperature (60 to 70°C) or longer thermocoagulation time is necessary to reach an optimal lesioning effect. However, further comparative studies, such as prospective, randomized, case-controlled, double-blind studies are necessary to verify this observation.

Single-shot echoplanar imaging (SS-EPI) has been used in most diffusion imaging studies, including all TN studies with DTI. However, SS-EPI is very sensitive to susceptibility artifacts; it requires long echo trains and echo time to encode whole *k*-space within 1 echo signal intensity, and the T2/T2* decay during acquisition causes image blurring and phase shift accumulation from field inhomogeneity, leading to geometric distortion [25]. Such effects are particular important for diffusion imaging near the skull base, which is prone to strong susceptibility artifact and field inhomogeneity. In this study, we used a RS-EPI with parallel imaging (GRAPPA) for DTI imaging. RS-EPI can reduce acquisition time by partitioning the *k*-space into segments along the readout direction, and parallel imaging can reduce the echo train length, thus reducing image distortions with higher resolution and reducing blurring from T2/T2* signal intensity decay compared with SS-EPI [26]. The superiority of RS-EPI has been demonstrated for diffusion imaging of areas that are prone to strong susceptibility artifacts, such as the pediatric brain [27], breast [28], and craniovertebral junction [29]. The application of RS-EPI DTI in the cisternal segment of CN V was confirmed in this study.

We recognize there are some limitations to this study. First, the partial volume effect, especially from imaging voxels with the cerebrospinal fluid (CSF) signal, might lead to error in DTI measurement. In general, there are three options to overcome this problem. 1) Use a DTI sequence with higher spatial resolution; however, this requires a very long scan time and is not suitable for a clinical study. 2) Regress out the voxels with the CSF signal, or by tissue segmentation. This is often used in modern neuroimaging studies; however, its use for a specific cranial nerve in the skull base surrounded by CSF has not been validated. 3) Define a threshold of white matter, such as a FA value > 0.2, and exclude all voxels with a FA below this limit. This may work in normal white matter, but will exclude white matter with severe demyelination or axonal injury. In this study, we co-registered DTI images to MP-RAGE and selected the imaging voxels in the axial slice containing the most voxels of CN V. Each voxel can be checked simultaneously in both DTI and MP-RAGE images to make sure the voxel is within the CN V. Furthermore, choosing the voxels in the slice containing the most voxels of CN V may reduce the partial volume effect in cranial-caudal images. Other limitations include that the study population was small, and the disease duration differed between patients. Long-term follow-up of patients to evaluate the prognostic value of DTI metrics and pain outcomes is necessary.

## Conclusions

Our results suggest demyelination and decreased CN V volume are the major microstructural abnormalities in TN caused by NVC. DTI may have the potential for predicting response to radiofrequency rhizotomy, but further investigation is necessary. Further studies are needed to understand how DTI metrics can quantitatively represent the pathophysiology of TN, and to examine the application of DTI in the treatment of TN.
